# Chemical Composition, Antimicrobial and Antioxidant Activity of Essential Oil from *Allium tenuissimum* L. Flowers

**DOI:** 10.3390/foods11233876

**Published:** 2022-12-01

**Authors:** Meiping Li, Xiying Zhao, Manjun Xu

**Affiliations:** School of Life Science, Shanxi University, Taiyuan 030006, China

**Keywords:** *Allium tenuissimum* L. flowers, essential oil, chemical composition, antimicrobial, antioxidant activity

## Abstract

*Allium tenuissimum* L. as a kind of food condiment in northern China, is popular among more and more consumers owning to its special flavor from the flower. However, its composition has not been widely studied. Hence, the aim of this study was to investigate the chemical composition and antimicrobial and antioxidant activity of essential oil from *Allium tenuissimum* L. flowers. Gas chromatography–mass spectrometry (GC-MS) was applied to detect the chemical composition. The antimicrobial activity against foodborne pathogens was evaluated by measuring the zones of inhibition (ZOI), the minimal inhibitory concentration (MIC), and the minimal bactericidal concentration (MBC). The antioxidant effect was tested by the scavenging capacity on DPPH, ABTS^+^•, and •OH. The results of GC-MS showed that 72 volatile components were isolated and the structures 68 of them were identified, which comprised about 91.92% of the total composition of the oil. Among these compounds, terpenoid compounds and sulfurous compounds had the highest contents, especially dimethyl trisulfide. Our investigation demonstrated that the essential oil has better antimicrobial efficiency to *Staphylococcus aureus*, *Bacillus subtilis*, *Escherichia coli, Aspergillus flavus*, and *Saccharomyces cerevisiae*. In addition, the essential oil had a strong stability to UV. Furthermore, the essential oil exhibited a high radical-scavenging effect on DPPH, ABTS^+^•, and •OH, which is significant for application in the food industry. In conclusion, the essential oil could be used as an inexpensive and natural antibacterial and antioxidant agent in food.

## 1. Introduction

*Allium tenuissimum* L. is a perennial herb belonging to the allium in the Amaryllidaceae family, which is known as Tianxiang flowers and Mama flowers in China, mainly distributed on slopes, grasslands, or dunes in altitudes below 2000 m in Shanxi, Heilongjiang, Jilin, Liaoning, and Shandong provinces etc. It is also found in Russia’s Siberia and Mongolia [1]. It is an excellent plant for windbreak and sand fixation, soil and water conservation, and can be easily grown whether in desolate cold sand or drought areas. It is not only a good kind of herbage but also a type of detoxification plant. The local herdsmen often feed *Allium tenuissimum* L. when livestock ingest poisonous weeds. At the same time, the *Allium tenuissimum* L. flowers is a good ornamental plant attributing to having white or lilac flowers and long flowering period. The inflorescence and seeds of *Allium tenuissimum* L. are edible. The flowers are specially designed for flavoring and present a special and rich flavor, which is better than green onions and garlic, especially after frying. Its leaves can also be used as a vegetable for spring and summer, which can be used in cooking, fried food, and cold mixes, and is full of delicious taste. In addition, its essential oil has been extracted to make pasta seasoning.

In recent years, food safety has been paid more and more attention. Due to the undesirable problems and side effects that arise from the consumption of artificial chemical compounds, the use of chemical preservatives is believed to have harmful side effects to consumers, so it is very important to seek safer food preservation methods. On the other hand, plant essential oils play an important role in controlling biological deterioration [2]. Essential oils which were extracted from a variety of spices, herbs, and plants showed antimicrobial activity against bacteria and fungus [3,4,5,6,7]. According to Ragina [8], white wormwood, rose-scented geranium, and bay laurel essential oils prevent Bacillus typhi murium and *Escherichia coli*. Miroslava [9] showed that coriander essential oil has the most inhibitory activity against B. subtilis. According to Youssef [10], chrysanthemum essential oil showed significant antibacterial activity against Streptococcus agalactiae, with a MIC value of 62.5 g/mL. Luo [11] showed that oregano essential oil (OEO) was also effective in killing Vibrio vulnificus in oysters at 25 °C, decreasing bacteria numbers by 48.2% after 10 h of treatment with 0.09% OEO. These essential oils are natural alternatives to extending the shelf life of the products. They are used in cosmetics, medicine, and the pharmaceutical industry extensively [12,13]. Researchers thought the existence of terpenoid compounds was the main reason that they had well-known antimicrobial activity. Compared with chemical components of some Allium genus essential oils, the literature about the antimicrobial and antioxidant potential of *Allium tenuissimum* L. is relatively small [14,15,16,17,18].

Garlic belongs to the Allium genus and its essential oil contains abundant sulfur compounds such as methylallyl trisulphate, diallyl disulphide, and diallyl trisulphide [19,20,21]. The antimicrobial biological activity of garlic was attributed to the high content of these bioactive organosulfur compounds [22]. According to our studies [23], the main volatile components were identified as dimethyl sulfide, dimethyl disulfide, dimethyl trisulfide, and 3-methyl-butanal. These substances are also high in essential oil and have quite similar phytochemical and nutraceutical effects as plants of the Allium genus [24,25]. So far, although there have been numerous investigations on the chemical compositions and antimicrobial activity of Allium [26,27], there are only two studies on its chemical composition, and no literature on the antimicrobial and antioxidant activity of the essential oil from the *Allium tenuissimum* L. flowers [1,23,24,25].

Oxidation is one of the most common corruption mechanisms of compounds and antioxidants have been widely used; however, with the development of the times, consumers are more keen on all-natural oxidants. Many scholars are increasingly paying attention to extracting low-toxic, safe, and efficient antioxidants from plants to meet people’s daily needs, and essential oils are good choices and can be used as an alternative to synthetic antioxidants. Therefore, there are many studies on the antioxidant properties of plants at home and abroad. Djamel [28] suggests that essential oils can prolong lipid stability and that citrus essential oils can effectively reduce lipid oxidation in sardines. Irene [29] confirmed that parsley essential oil had the highest inhibition on DPPH radicals and FRAP. Many researchers [30,31,32,33,34] indicated that the original essential oils had the highest antioxidant activity. Gyung-Rim [35] extracted essential oils from Maclura triscuspidata fruit, which had good natural antioxidant potential. The essential oils obtained by Lee [36] by CO_2_ supercritical extraction have obvious antioxidant properties. Researchers have applied the antioxidant properties of volatile oils extracted from different plants to food, beauty, medicine, and more.

Therefore, the purpose of our study was to analyze the composition and evaluate the antimicrobial activity against bacteria (*Bacillus subtilis*, *Staphylococcus aureus*, and *Escherichia coli*) and fungus (*Saccharomyces cerevisiae* and *Aspergillus flavus*) of the essential oil. To further explore whether the essential oil has antioxidant properties, the scavenging capacity against DPPH, ABTS^+^•, and •OH was examined as well. The study offers some experimental data for the understanding and application of *Allium tenuissimum* L.

## 2. Materials and Methods

### 2.1. Plant Materials and Microbial Strain

*Allium tenuissimum* L. flowers were collected from Shanxi Province of China in August 2019. The flowers were air-dried at room temperature (20–32 °C). Three bacteria (*Bacillus subtilis*, *Staphylococcus aureu*, and *Escherichia coli*) and two fungus strains (*Aspergillus niger* and *Saccharomyces cerevisiae*) used for this study were obtained from the microbiology laboratory of Shanxi University at Taiyuan, Shanxi Province, China. The strains were cultured at 37 °C/28 °C on nutrient agar (NA)/nutrient broth (NB) media.

### 2.2. Extraction of Essential Oil

The dried *Allium tenuissimum* L. flowers were ground and sieved through 80 mesh size. Then, 150 g of the samples was subjected to 1200 mL distilled water, soaked overnight for 12 h, and then hydrodistilled for 4 h and submerged for 30 min using a Clevenger apparatus. The oily layer obtained on top of the aqueous distillate was separated by anhydrous ether and dried with anhydrous sodium sulfate. The oil obtained was stored in tightly closed dark vials at 4 °C until further analysis. Its extraction yield was calculated as Equation (1) [37].

(1) $$\mathrm{yield}\;\mathrm{of}\;\mathrm{oil}\%=\frac{\mathrm{Weight}\;\mathrm{of}\;\mathrm{oil}}{\mathrm{Weight}\;\mathrm{of}\;\mathrm{Allium}\;tenuissimum\;L.\;\mathrm{flowers}}\times \;100$$

### 2.3. Identification of the Components of Essential Oil

The GC-MS analysis of essential oil was carried out using a 7890 gas chromatograph interfaced to a 5975C mass selective detector (Agilent Technologies, Palo Alto, CA, USA). The chromatograph separation was operated on a HP-5MS chromatographic column (30 m × 0.25 mm, 0.25 mm film thickness, Agilent) using helium as the carrier gas at a constant flow rate of 1 mL min^−1^. The oven temperature was held 35 °C for 4 min, programmed to 150 °C at a rate of 2.5 °C/min, and finally raised to 300 °C at 10 °C/min. The volume of the 0.4 µL essential oil sample was injected manually by 10 microliter syringes in a split mode ratio of 1:20 at 250 °C injector temperature. The solvent delay was 2 min. The transfer line temperature was set at 280 °C. The mass detector was operated in electron impact mode with the energy of 70 eV and scanning range from m/z 30 to 500. The quadrupole and ion source were 150 and 230, respectively.

The components were identified by comparing the obtained retention indexes (RI) using a homologous series of n-alkanes (C7-C40) under the same operating conditions and literature [38] while matching their mass spectra with NIST11 mass spectral library. The abundance of each compound was expressed on the relative percentage of the total area of the chromatogram.

### 2.4. Evaluation of Antimicrobial Activities of Essential Oil

#### 2.4.1. Preparation of the Microbial Strains

The three bacteria included two Gram-positive (*Bacillus subtilis* and *Staphylococcus aureus*) and one Gram-negative (*Escherichia coli*). Referring to the literature [39], the three bacteria were grown in beef extract peptone medium (3 g beef extract powder, 10 g peptone, and 5 g NaCl dissolved in 1000 mL distilled water; 140 r/min shake culture; pH was 7.0–7.2 adjusted by NaOH; autoclaved at 121 °C for 20 min) at 37 °C for 24 h before use. The two fungi were *Saccharomyces cerevisiae* and *Aspergillus flavus* grown in potato dextrose agar (PDA) medium (200 g potatoes and 20 g glucose dissolved in 1000 mL distilled water; 160 r/min shake culture; pH naturally; autoclaved at 121 °C for 20 min) at 28 °C respectively for 48 h and 72 h (*Aspergillus flavus* need longer time to reproduce to a certain extent in quantity than *Saccharomyces cerevisiae*).

After be activated in liquid medium, five microbial strains were respectively diluted to 10, 10^2^, 10^3^, 10^4^, 10^5^, 10^6^, 10^7^, and 10^8^ times by sterile saline water (0.9%). A 200 mL microbial suspension was added to the solid medium and incubated to generate some colonies at the appropriate temperature. Then, the dilution ratio of the microbial suspension was determined by dilution method of plate counting and the final concentration of the microbial suspension used in this study was 10^6^–10^7^ cells/mL (experiments ensured reproducibility and counted colonies from 30 to 300 on the plates).

#### 2.4.2. Agar Diffusion Method

The antimicrobial activity of *Allium tenuissimum* L. flowers essential oil was tested by using the agar diffusion method reported by Liu and Fu [40,41] with some modifications. Sterilized paper discs (6 mm diameter) were impregnated with different concentrations of essential oils in ethyl ether for 20 min (100%, 50%, 25%, 12.5%, 6.25%, 3.13%, and 1.57% *v*/*v*) and impregnated with ethyl ether of the blank control group. Then, the paper discs were taken out for evaporating the ethyl ether and placed onto nutrient agar. The upside-down plates were incubated at 35 °C for 40 h to obtain bacteria and 28 °C for 60 h to obtain fungus. The result of antimicrobial activity was evaluated by measuring the diameters of inhibition zones (mm) in millimeters including the paper discs’ diameter and recorded in triplicate.

#### 2.4.3. Minimum Inhibitory Concentration (MIC), Minimum Bactericidal Concentration (MBC), and Minimum Fungicidal Concentration (MFC) Assay

Five different strains obtained from the pre-culture (three bacteria for 24 h, two fungi for 48 h/72 h) were adjusted to 10^6^–10^7^ cells/mL by the dilution method of plate counting. The minimal inhibitory concentration(MIC) of the essential oil extracted from *Allium tenuissimum* L. flowers was determined using the microdilution broth method in 96-well microplates [42,43,44]. A detailed account is as follows: 100 mL essential oil was diluted to different concentrations in a Tween-80 1% *v/v* aqueous solution (5%, 2.5%, 1.25%, 0.63%, 0.32%, and 0.16%) adopting two-fold dilutions with nutrient broth. A 100 μL standard suspension of the microorganisms was absorbed and added into the well containing essential oil, and no essential oil was used as the blank control. Finally, all microtiter plates were incubated (three bacteria at 35 °C/20 h and two fungi at 28 °C/40 h). The MIC was determined as the lowest concentration until it visually inhibited microbial growth [44]. After broth microdilution, 20 µL of the sample from wells that were having no visible microbial growth was sub-cultured in agar nutrient plates. Subsequently, three bacteria at 35 °C/20 h and two fungi at 28 °C/40 h were incubated. Minimum Bactericidal Concentration (MBC) and Minimum Fungicidal Concentration (MFC) was determined as the lowest concentration able to kill the 99% of microbial colonies on agar nutrient plates [45]. All the experiments were performed in triplicate.

### 2.5. Time Kill Assay

A time kill test was accomplished with the strain which was the most sensitive to essential oil, and the strain was chosen according to the experimental results of the inhibition zone diameters, MIC and MFC. The microbial suspension concentration was adjusted to 106–107 cells/mL. A 500 μL sample of several various concentrations (MFC, 2MIC, MIC, and 1/2MIC) of essential oil and 500 μL of tested microbial suspension were added respectively to 24-well microplates [46]. In the last well, 500 μL of microbial suspension and 500 μL of nutrient broth were added as the blank control group. A 50 μL sample of microbial suspension in the microplates was removed after incubating 0 h, 1 h, 2 h, 4 h, 8 h, 12 h, 24 h, and 30 h, and then certain multiples were diluted with saline and sub-cultured in agar nutrient plates in triplicate to observe the process of inhibiting *Saccharomyces cerevisiae*.

### 2.6. The Effect of pH Value and Ultraviolet Irradiation on the Antimicrobial Activity of Essential Oil

The pH value of the medium was adjusted to five gradients (4.0, 5.0, 6.0, 7.0, and 8.0) [47]. The blank control group of natural pH value was 6.0. Various exposure time periods (20 min, 40 min, 60 min, and 80 min) were given under an ultraviolet lamp in order to investigate the effect on the antimicrobial activity of essential oil. The essential oil that was not exposed to ultraviolet radiation was regarded as the blank control group. The diameters of inhibition zones for expressing the effect of pH value and ultraviolet irradiation on the antimicrobial activity were measured according to the method mentioned above. The concentration used in the above is 100%.

### 2.7. Antioxidant Activity of Volatile Oil In Vitro

#### 2.7.1. Determination of DPPH Clearance Rate

The clearance test of DPPH radicals was slightly modified according to the method of Lv [48]. An amount of 2 mL of volatile oil solution (3%, 2%, 1%, 0.5%, and 0.25%) was added to 2 mL 0.1 mmol/L of DPPH-methanol solution. As the positive control, 2% propylene gallate was used. After resting at room temperature for 30 min, the absorbance value A was measured by spectrophotometer at 517 nm. The absorbance value A_0_ was added to the sample with 2 mL methanol solution instead of 2 mL DPPH-methanol solution, and the absorbance value A_1_ was added to the sample with 2 mL methanol solution. The DPPH free radical scavenging rate is expressed by the following Formula (2):

(2) $$\mathrm{DPPH}\;\mathrm{clearance}\;\mathrm{rate}\%\;=\;\frac{{}_{A_{1}\;-\;A\;+\;A_{0}}}{A_{1}}\times \;100$$

#### 2.7.2. Determination of ABTS^+^ • Clearance Rate

The clearance experiment of ABTS^+^• was conducted by referring to the method of Ma [49] and the method was slightly modified. The 20 mL ABTS^+^• solution was mixed with 5.2 mL 2 mol/L potassium persulfate solution, and the volume was constant to 100 mL in methanol solution. The solution was placed at room temperature and away from light for 12 h. An amount of 3.9 mL of the above mixed solution and 0.1 mL of methanol diluted essential oil solution (1%, 2%, 3%, 4%, and 5%) were mixed evenly and placed at room temperature under dark conditions for 40 min. The absorbance value A_1_ was measured using methanol as a reference solution. The absorbance value measured by 0.1 mL methanol solution instead of essential oil dilution was A_0_. The ABTS^+^• clearance rate was expressed by the following Formula (3):

(3) $${\mathrm{ABTS}}^{+•}\;\mathrm{clearance}\;\mathrm{rate}\%\;=\;\frac{A_{0}-A_{1}}{A_{0}}\times \;100$$

#### 2.7.3. Determination of •OH Clearance Rate

The hydroxyl radical scavenging experiment is based on the experiment of Li et al. [50], with some modifications as follows. Take 2 mL of methanol-diluted essential oil solutions with different concentrations (1.25%, 1%, 0.75%, 0.5%, and 0.25%). Then, add 2 mL 1.5 mmol/L FeSO_4_, 2 mL 1.5 mmol salicylic acid, and 2 mL H_2_O_2_, respectively. After the mixture is evenly mixed, the absorbance lll value is measured at 510 nm by UV spectrophotometer with methanol solution as the reference solution and marked as A_1_. Under the same conditions, the absorbance value of the 2 mL sample solution is replaced by 2 mL methanol solution and marked as A_0_. The absorbance value measured by replacing 2 mL salicylic acid with 2 mL methanol solution is denoted as A_2_, and the hydroxyl radical clearance rate is calculated as follows (4):

(4) $$•\mathrm{OH}\;\mathrm{clearance}\;\mathrm{rate}\%\;=\;\frac{A_{0}-A_{1}+A_{2}}{A_{0}}\times \;100$$

The results of each group of tests were measured three times in parallel and averaged.

## 3. Results and Discussion

### 3.1. Chemical Composition of Essential Oil

The essential oil was obtained as a light yellow transparent liquid and had a specific garlicky aroma. The yield of essential oil is 0.13%. The chemical compositions of essential oil were analyzed by GC-MS. The analysis result is presented in Table 1.

According to the GC-MS analysis, a total of 72 components in the essential oil were isolated and the structures of 68 of them were identified, which comprised about 91.92% of the total composition of the oil. The major compounds of the essential oil were 19 sulfurous compound (58.58%), 14 terpenes (12.80%), 9 hydrocarbons (1.78%), 6 esters (2.40%), 7 alcohols (3.83%), 6 aldehydes (2.71%), 3 ketones (2.11%), and 4 other compounds (7.71%). The essential oil was characterized as sulfide compound (38.91%) and terpenes (12.80%) being predominant. The components with a percentage of more than 4% are: dimethyl trisulfide (21.16%), 1,3-dithiane (11.94%), 1,8-cineole (6.32%), dimethyl disulfide (4.47%), 2-camphanonoe (4.91%), and 1-propenyl trisulfide (5.82%). These are the main components of *Allium tenuissimum* L. flowers essential oil. Most of these components are present in many other essential oils such as garlic and chives [51,52]. The main sulfur compound is dimethyl trisulfide in *Allium tenuissimum* L. flowers essential oil. It is different from garlic essential oil, of which the main components are Diallyl trisulfide and Diallyl disulfide [53]. The presence of a sulfurous compound in a significant amount in the oil is in agreement with our previous work [23], indicating that the occurrence of a sulfurous compound as the major constituent may be a characteristic of the *Allium tenuissimum* L. flowers essential oil.

### 3.2. Antimicrobial Activity of Essential Oil

The presence or absence of inhibition zone diameters was used to qualitatively assess the antimicrobial activity. The results of antimicrobial activity of *Allium tenuissimum* L. flowers essential oil using diffusion method on agar (solid diffusion assays) are shown in Table 2.

According to the results of the diameter of inhibition zones (Table 2), it is not hard to see that five tested microbial strains showed sensitivity to the essential oil. However, the degree to inhibit the growth of each strain was different as it was dependent on microorganism strain, inhibition time, and essential oil concentration. For three bacteria, when the suppression time was within 20 h, the essential oil demonstrated strong antimicrobial activity against *Bacillus subtilis* with inhibition zones of more than 13.5 mm at 25% concentration. The essential oil showed moderate activity against *Staphylococcus aureus* with inhibition zones 6.0–13.5 mm and *Escherichia coli* with zones 6.0–13.0 mm at concentrations of 6.25–100% (*v*/*v*). For two fungi, when the suppression time was within 40 h, *Saccharomyces cerevisiae* had inhibition zones more than 19.8 mm at concentrations of 1.57–100% (*v*/*v*), followed by *Aspergillus flavus* (10.8–20.0 mm). The whole plate showed no *Saccharomyces cerevisiae* growth at concentrations greater than 12.5% (*v*/*v*), indicating that *Saccharomyces cerevisiae* was the most susceptible to *Allium tenuissimum* L. flowers essential oil in five tested strains. In addition, with the decrease in the essential oil concentration, the antimicrobial activity decreased. As can be seen from Table 2, the antimicrobial activity decreased and the antimicrobial effect of various concentration essential oils on various strains with time increasing. According to the reports [3,54] about inhibition zone diameter results as follows: zone diameters equal to 6 mm or below, not sensitive; between 6 and 10 mm, sensitive; between 10 and 15 mm, medium sensitive; between 15 mm and 20 mm, very sensitive; and equal to or larger than 20 mm, extremely sensitive. As indicated in Table 2, *Allium tenuissimum* L. flowers essential oil exhibited a certain antimicrobial activity against the tested fungi and Gram-positive bacteria. However, the antimicrobial activity of the tested essential oil is slightly lower than Allium essential oil.

In order to assess whether the essential oil has bacteriostatic or bactericidal and fungicidal effects on the five tested strains at 0.16–5% *v*/*v*, the MIC and MBC/MFC values were measured and the results are shown in Table 3. The best activities were observed against *Saccharomyces cerevisiae* with an MIC value of 0.32% *v*/*v* and an MFC value of 0.63% *v*/*v* (Table 3) with the disk diffusion method. The results demonstrated that the essential oil was most resistant to *Saccharomyces cerevisiae* among the five tested strains. This is consistent with the result of the agar diffusion test in Table 2. However, it did not have significant activity against *Escherichia coli* (MIC 2.5% *v*/*v*, MBC 5% *v*/*v*) and *Bacillus subtilis* (MIC 1.25% *v*/*v*, MBC > 5% *v*/*v*). This indicates that *Bacillus subtilis* was entirely killed by the oil at concentrations less than 5%*v*/*v*. The reason may be due to the cell wall structures and components of the strains being different, causing the essential oil to affect the strains in different ways. Therefore, the essential oil exhibited different inhibitory effects on different strains. The content of sulfide compound and terpenes is predominant, which plays an important role in the antibacterial effect. The specific antibacterial mechanism needs further study.

### 3.3. Time-Kill Assay

**Figure 1:**
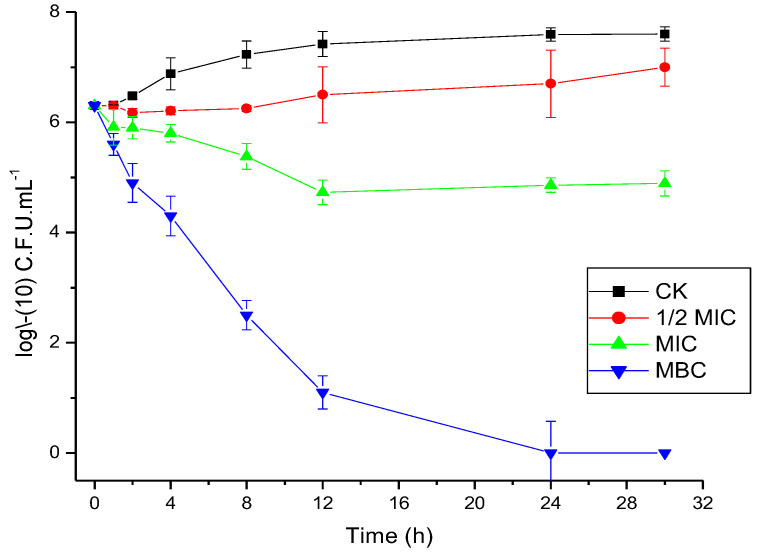
Time−kill assay of essential oil to *Saccharomyces cerevisiae* at concentrations of 0% *v*/*v* (CK), 0.16% *v*/*v* (1/2MIC), 0.32% *v*/*v* (MIC), and 0.64% *v*/*v* (MFC) and the control. Data represent the mean of three independent experiments ± sd.

According to the experimental results above, *Allium tenuissimum* L. flowers essential oil has strong antimicrobial activity against *Saccharomyces cerevisiae*. To further confirm the dynamic process of sterilization and explore whether the essential oil has potency in food sterilization and as a preservative, the time courses of *Saccharomyces cerevisiae* growth by virtue of different concentrations of essential oil were plotted. As shown in Figure 1, the influence of the essential oil to inhibit the growth of *Saccharomyces cerevisiae* was quite clear. When the concentration reached a concentration of MFC (0.64% *v*/*v*) for 24 h, the *Saccharomyces cerevisiae* stopped reproducing. However, the phenomenon was not obvious in 0.5 MIC and MIC concentrations of essential oil. On the other hand, the data showed that the various sensitivity of *Saccharomyces cerevisiae* to the tested essential oil was related to concentration and time. With the concentration of essential oil increasing, the inhibition of essential oil to *Saccharomyces cerevisiae* was more and more significant. These findings demonstrated that the antimicrobial effect of essential oil from *Allium tenuissimum* L. flowers had the relation of a dose-dependent manner and increased with the dose. As can be seen from the time-kill assay, the number of bacteria decreased with the increase in concentration, indicating that the antibacterial effect becomes stronger. However, the effect did not increase with the extension of time, so the antibacterial effect displayed a strong concentration dependence and a low time dependence.

### 3.4. Stability of Essential Oil

The results of the effect of pH and ultraviolet irradiation on the antimicrobial activity of essential oil are shown in Table 4 and Table 5, respectively.

As shown in Table 4, it was apparent that the microorganism of the control group (non-essential oil) can grow normally at pH 4.0–9.0. After adding essential oil, the inhibition zone gradually decreased with the increase in pH value, which accounted for the antimicrobial effect of *Allium tenuissimum* L. flowers essential oil against the tested five strains gradually reducing. In conclusion, the antimicrobial effect of the essential oil was related to the pH value of the medium. The lower the pH value, the higher the antimicrobial activity, which may be concerned with the structural form of essential oil active constituents and the decreased personal activity of the tested strains in the acid environment.

As can be seen from Table 5, no significant changes in the antimicrobial activity of the essential oil against the tested strains were observed before and after UV irradiation at different times. The results indicate that *Allium tenuissimum* L. flowers essential oil has a strong stability to UV. Ultraviolet sterilization is applied to the processing of many foods, so one of the important characteristics for natural preservatives is good UV stability.

### 3.5. Antioxidant Activity of Volatile Oil In Vitro

#### 3.5.1. The Clearance Rate of DPPH

**Figure 2:**
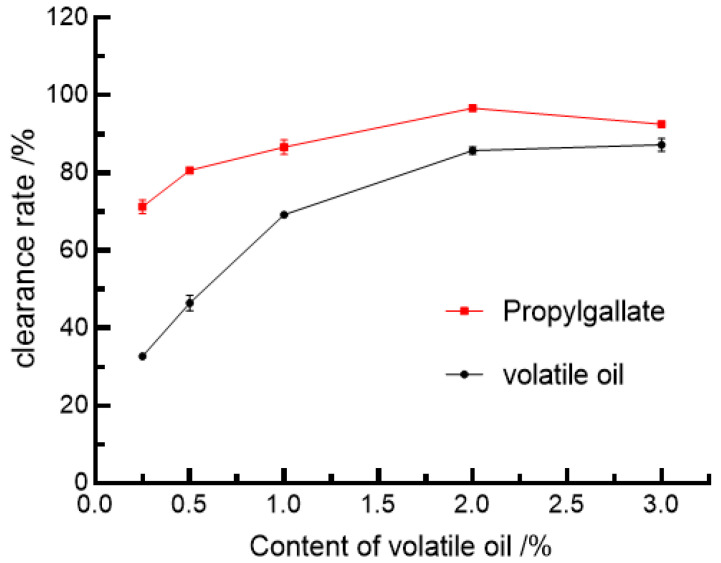
DPPH scavenging activity of *Allium tenuissimum* L. flowers essential oil.

The principle behind the scavenging of DPPH radicals by in vitro antioxidant experiments with volatile oils is that DPPH, also known as 1,1-diphenyl-2-trinitrophenylhydrazine, is a very stable radical centered on unpaired single electron nitrogen attached to the space barrier single electron pairing of the three benzene rings from resonance stabilization, with which it forms a purple complex when dissolved in alcohol solutions, with maximum absorption at 517 nm. It has a maximum absorption intensity of 517 nm. When volatile oils are present with DPPH scavengers, the color development reaction is weakened by the provision of hydrogen to trap single electrons on DPPH [55]. The ability to scavenge free radicals is evaluated by the lightening of the color of the reaction solution when the binding capacity is high. The scavenging effect of volatile oils on DPPH radicals is shown in Figure 2.

As can be seen from Figure 2, the scavenging effect on DPPH increased with increasing concentration of volatile oil and its antioxidant capacity continued to strengthen but was lower than that of the positive control, propyl gallate. When the concentration of volatile oil was greater than 1%, the rate of increase in DPPH scavenging slowed down. At a volatile oil concentration of 0.5%, DPPH was cleared by approximately 50%.

#### 3.5.2. The clearance Rate of ABTS^+^•

**Figure 3:**
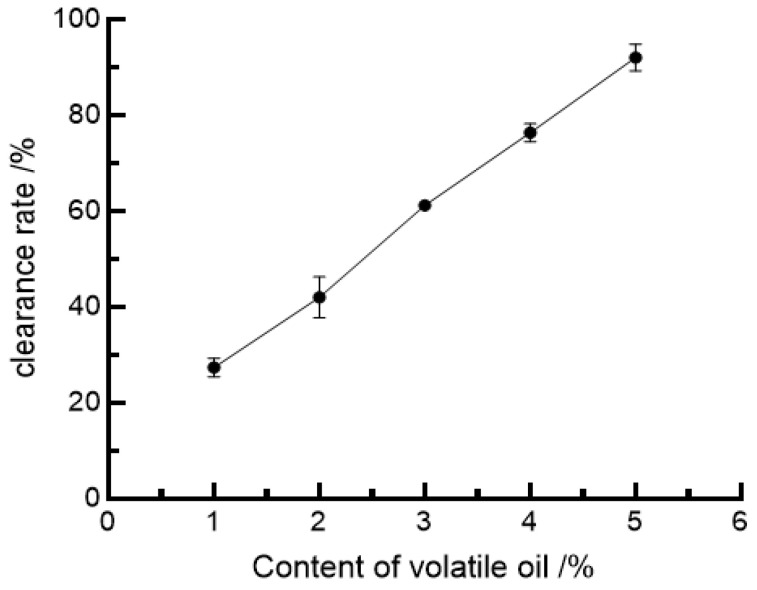
ABTS^+^• scavenging activity of *Allium tenuissimum* L. flowers essential oil.

Under the action of certain oxidants, ABTS^+^• is oxidized to the green ABTS^+^•, which has maximum light absorption at 734 nm. When antioxidants are present, the conversion of ABTS^+^• is inhibited, thus weakening the ability of the chromogenic reaction [56] to evaluate the ability to scavenge ABTS^+^• capacity. The scavenging of ABTS^+^• by the volatile oil of Leptospermum album is shown in Figure 3.

As shown in Figure 3, with the increase in volatile oil concentration of fine leek flower, the scavenging rate of ABTS^+^• increased positively, and in the range of volatile oil test concentration (1–5%), the scavenging rate of ABTS^+^• was linearly related to the change in volatile oil concentration, and the regression equation was Y = 16.315X + 12.425, R_2_ = 0.9884, when the volatile oil concentration was 2.3%. The regression equation was Y = 16.315X + 12.425, R_2_ = 0.9884, and the removal rate of ABTS^+^• reached 50% at 2.3% volatile oil concentration.

#### 3.5.3. The Clearance Rate of •OH

**Figure 4:**
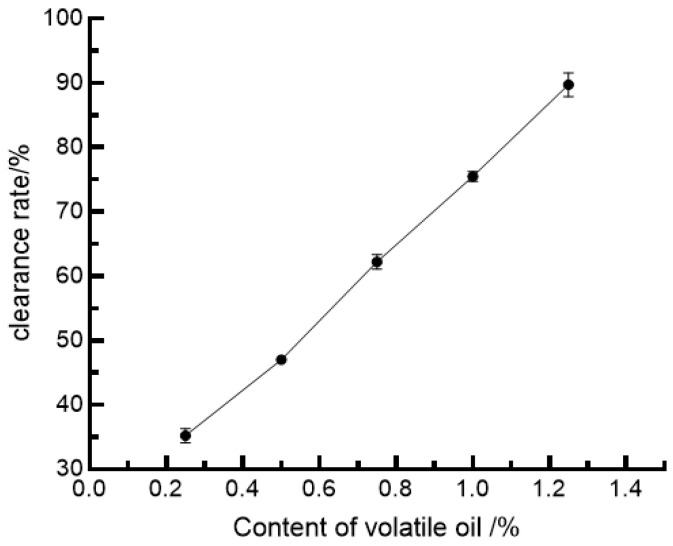
•OH scavenging activity of *Allium tenuissimum* L. flowers essential oil.

The principle of hydroxyl radical scavenging by the volatile oil of Lepidium leucocephala: the •OH produced by the Fenton reaction H_2_O_2_ + Fe^2+^ = •OH + H_2_O + Fe^3+^ reacts with salicylic acid to produce 2,3-dihydroxybenzoic acid with maximum absorption at 510 nm. If a reaction scavenger of •OH is present in the volatile oil, it reduces the amount of •OH produced by the Fenton reaction, thus lightening the color of the colored solution as a means of evaluating the ability to scavenge hydroxyl radicals [57]. The scavenging effect of volatile oils on hydroxyl radicals (•OH) is shown in Figure 4.

As shown in Figure 4, there was a positive and linear correlation between the volatile oil content and the scavenging of hydroxyl radicals, with a regression equation of Y = 53.948X + 20.9390000 and R_2_ = 0.9859, giving a 50% scavenging of hydroxyl radicals at a volatile oil concentration of 0.54%.

## 4. Conclusions

In conclusion, experimental data from the present study indicated that the essential oil possesses high levels of terpenoid compounds and sulfurous compounds (58.58%), especially sulfide compound (38.91%). Moreover, the essential oil had significant antimicrobial activity against fungi and Gram-positive bacteria than Gram-negative bacteria. When the concentration was greater than 12.5% (*v*/*v*), the whole plate showed no *Saccharomyces cerevisiae* growth. In general, essential oils are more effective inhibitors of fungi than of bacteria [58,59], which is consistent with the conclusion reached in our experiment. Meanwhile, the antimicrobial activity increased with increased essential oil concentration and increased treatment time, and was also related to the pH value of the medium. In addition, the essential oil had a strong stability to UV. The volatile oil showed some antioxidant activity against DPPH radicals, ABTS^+^•, and hydroxyl radicals, and was positively correlated with the volatile oil concentration. The scavenging rate of DPPH was not linearly related to the concentration of volatile oil and the antioxidant activity was lower than that of the positive control, propyl gallate, while the scavenging rate of ABTS^+^• and hydroxyl radicals was linearly related to the concentration of volatile oil.

To the best of our knowledge, this is the first study that shows chemical composition and evaluation of antimicrobial and antioxidant activity for *Allium tenuissimum* L. flowers essential oil. Our research provides a reasonable study to explore whether the *Allium tenuissimum* L. flowers essential oil has potential for utilization as a natural alternative to traditional food preservatives in food and pharmaceutical industries to enhance food safety and shelf life. In conclusion, *Allium tenuissimum* L. flowers essential oil is a cheap but favorable resource with strong antimicrobial capacity. The study provided a theoretical basis for the potential utilization of the tested essential oil in food and pharmaceutical industries. However, further research on the mechanisms of action and the effect on other food spoilage and poisoning microorganisms are still necessary to gain a complete understanding in order to justify real applications of *Allium tenuissimum* L. flower essential oil in food practices as a natural antimicrobial agent.

## Figures and Tables

**Table 1:** GC-MS analytical results of composition for the *Allium tenuissimum* L. flowers essential oil.

NO	Compounds	Relative Content/%	RI	
			Calculated Value	Literature Value
1	Methyl thiirane	0.13%	605	619
2	Allyl sulfhydrate	0.36%	665	583
3	Isovaleraldehyde	0.24%	679	632
4	Methyl ethyl disulfide	0.43%	684	838
5	Pentanal	0.09%	740	715
6	Isopentanol	0.07%	760	744
7	Dimethyl disulfide	4.47%	761	747
8	Hexanal	0.30%	819	804
9	2-Ethyl-trans-2-butenal	0.08%	844	791
10	2,4-Dimethyl thiophene	0.09%	877	878
11	3,4-Dimethyl thiophene	1.95%	903	867
12	Allyl methyl disulphide	0.82%	914	-
13	1,3-Dithiane	4.52%	927	1002
14	Methyl propyl disulfide	0.78%	929	920
15	Isomer of 1,3-Dithiane	7.42%	936	1002
16	Camphene	0.33%	944	935
17	Dimethyl trisulfide	21.16%	963	954
18	Sabinen	0.19%	971	971
19	2-Pentylfuran	0.40%	991	991
20	p-Cymene	0.55%	1018	1022
21	1,8-cineole	6.32%	1023	1022
22	γ-Terpinene	0.65%	1054	1282
23	Terpinolene	0.17%	1084	1084
24	Diallyl Disulfide	0.19%	1096	1079
25	Linalool	1.24%	1099	1099
26	Tanacetone	1.13%	1101	1124
27	Nonanal	1.42%	1104	1096
28	Thujone	0.82%	1106	1097
29	2,4,5-Trithiahexane	1.10%	1115	1110
30	3,5-Dimethyl-1,2,4-trithiolane	0.58%	1120	1127
31	1-propenyl trisulfide, (Z)-	1.09%	1127	1136
32	2-Camphanone	4.91%	1134	1136
33		3.22%	1141	
34	1-propenyl trisulfide, (E)-	4.73%	1151	1145
35	Borneol	1.82%	1158	1155
36	-	1.74%	1161	
37	Nerol	1.09%	1165	1204
38	Terpinen-4-ol	1.82%	1171	1182
39	α-Terpineol	0.51%	1187	1187
40	Methyl, tetrasulfide	4.14%	1201	1202
41	Cyclobutaneethanol, 1-methyl-2-(1-methylethenyl)-, cis-	0.52%	1208	1183
42	Propionic acid, 3-(isobutylthio)-	2.28%	1247	1260
43	bornyl acetate	1.05%	1282	1284
44	Lavandulyl acetate	0.85%	1291	1270
45	1.3,5-Diethyl-1,2,4-trithiolane	2.35%	1313	1307
46	Copaene	0.17%	1369	1368
47	-	1.17%	1383	
48	Caryophyllene	0.25%	1408	1408
49	-	1.95%	1462	
50	Varidiflorene	0.05%	1470	1471
51	ɑ-Selinene	0.59%	1478	1476
52	β-lonone	0.15%	1482	1486
53	Pentadecane	0.09%	1499	1512
54	Neryl propionate	0.14%	1510	1451
55	Spathulenol	1.15%	1564	1570
56	Caryophyllene oxide	0.56%	1569	1566
57	Hexadecane	0.13%	1598	1600
58	Cedrol	0.21%	1605	1589
59	β-Eudesmol	0.15%	1651	1628
60	Heptadecane	0.19%	1699	1700
61	Octadecane	0.19%	1799	1800
62	Fitone	0.58%	1837	1845
63	Nonadecane	0.07%	1899	1900
64	Methyl hexadecanoate	0.25%	1917	1925
65	Isophytol	0.20%	1942	1943
66	n-Hexadecanoic acid	0.42%	1957	1962
67	Ethyl hexadecanoate	0.09%	1994	1995
68	Linoleic acid, methyl ester	0.03%	2097	2092
69	Heneicosane	0.06%	2099	2100
70	Tricosane	0.65%	2295	2300
71	Tetracosane	0.04%	2395	2400
72	pentacosane	0.35%	2500	2500

Note: RI: retention index; -: unidentified.

**Table 2:** Antimicrobial activity of essential oil by Agar well diffusion method.

Essential Oil Concentration *v*/*v* (mL/mL)	Zones of Inhibition (mm) on Tested Microbial Strains									
	*Bacillus subtilis*		*Staphylococcus aureus*		*Escherichia coli*		*Aspergillus flavus*		*Saccharomyces cerevisiae*	
	20 h	40 h	20 h	40 h	20 h	40 h	40 h	60 h	40 h	60 h
100%	21.0 ± 0.57	16.2 ± 0.44	13.5 ± 0.40	12.3 ± 0.40	13.0 ± 0.35	11.3 ± 0.53	20.0 ± 0.15	14.2 ± 0.66	》	》
50%	17.3 ± 0.5	15.7 ± 0.35	12.3 ± 0.38	10.5 ± 0.38	10.8 ± 0.21	9.7 ± 0.38	16.5 ± 0.40	11.5 ± 0.31	》	》
25%	13.5 ± 0.36	10.5 ± 0.36	9.3 ± 0.45	8.2 ± 0.20	10.5 ± 0.36	9.5 ± 0.35	13.3 ± 0.31	10.0 ± 0.43	》	》
12.5%	10.8 ± 0.50	8.7 ± 0.49	8.7 ± 0.26	6.0 ± 0.06	9.8 ± 0.46	9.2 ± 0.25	11.0 ± 0.20	8.5 ± 0.21	》	》
6.25%	8.5 ± 0.36	8.3 ± 0.3	6.0 ± 0.06	6.0 ± 0.12	6.0 ± 0.00	6.0 ± 0.06	10.8 ± 0.21	8.0 ± 0.46	19.8 ± 0.51	19.0 ± 0.26
3.13%							9.3 ± 0.31	6.0 ± 0.12	18.2 ± 0.35	16.5 ± 0.2
1.57%							6.0 ± 0.00	6.0 ± 0.00	15.0 ± 0.51	14.8 ± 0.55
0%	6.0 ± 0.00	6.0 ± 0.00	6.0 ± 0.00	6.0 ± 0.00	6.0 ± 0.00	6.0 ± 0.00	6.0 ± 0.00	6.0 ± 0.00	6.0 ± 0.00	6.0 ± 0.00

Note: 》: the whole plate had no colonies; the diameter of the inhibition zones (mm), including the filter paper diameter (6 mm), are given as mean ± SD of triplicate experiments.

**Table 3:** Minimum Inhibitory Concentrations (MICs), Minimum Bactericidal Concentrations (MBCs), and Minimum Fungicidal Concentrations (MFCs) of *Allium tenuissimum* L. flowers essential oil.

	*Bacillus subtilis*	*Staphylococcus aureus*	*Escherichia coli*	*Aspergillus flavus*	*Saccharomyces cerevisiae*
MIC (% *v*/*v*)	1.25	0.63	2.5	0.63	0.32
MBC/MFC (% *v*/*v*)	>5	1.25	5	2.5	0.63

**Table 4:** Antimicrobial activities of *Allium tenuissimum* L. flowers essential oil against the tested microorganisms under different pH values.

Strains		Zone of Inhibition (mm) on Tested Microbial Strains					
		pH Value					
		4.0	5.0	6.0	7.0	8.0	9.0
*Bacillus subtilis*	contrast	regular growth	regular growth	regular growth	regular growth	regular growth	regular growth
	essential oil	25.6 ± 0.35	24.6 ± 0.20	22.9 ± 0.32	21.5 ± 0.40	19.2 ± 0.10	17.3 ± 0.25
*Staphylococcus aureus*	contrast	regular growth	regular growth	regular growth	regular growth	regular growth	regular growth
	essential oil	18.8 ± 0.35	16.3 ± 0.20	15.4 ± 0.47	13.6 ± 0.10	12.5 ± 0.35	10.6 ± 0.37
*Escherichia coli*	contrast	regular growth	regular growth	regular growth	regular growth	regular growth	regular growth
	essential oil	18.7 ± 0.26	16.3 ± 0.40	14.5 ± 0.26	13.0 ± 0.25	12.3 ± 0.21	10.6 ± 0.21
*Aspergillus flavus*	contrast	regular growth	regular growth	regular growth	regular growth	regular growth	regular growth
	essential oil	25.0 ± 0.26	22.7 ± 0.46	20.3 ± 0.47	18.2 ± 0.35	17.7 ± 0.46	16.0 ± 0.30
*Saccharomyces cerevisiae*	contrast	regular growth	regular growth	regular growth	regular growth	regular growth	regular growth
	essential oil	》	》	》	》	79.8 ± 0.42	70.5 ± 0.32

Note: 》: the whole plate had no colonies; the diameter of the inhibition zones (mm), including the filter paper diameter (6 mm), are given as mean ± SD of triplicate experiments.

**Table 5:** Antimicrobial activities of *Allium tenuissimum* L. flowers essential oil after ultraviolet radiation against the tested microorganisms.

Strains	Zone of Inhibition (mm) on Tested Microbial Strains				
	Ultraviolet Radiation Time (min)				
	20	40	60	80	Contrast
*Bacillus subtilis*	21.0 ± 0.38	21.3 ± 0.38	21.5 ± 0.21	21.6 ± 0.35	21.6 ± 0.20
*Staphylococcus aureu*	13.5 ± 0.10	13.5 ± 0.17	13.7 ± 0.30	12.8 ± 0.21	13.3 ± 0.10
*Escherichia coli*	13.4 ± 0.17	13.0 ± 0.43	13.6 ± 0.20	13.0 ± 0.43	13.2 ± 0.17
*Aspergillus niger*	20.1 ± 0.40	20.8 ± 0.53	19.9 ± 0.30	20.6 ± 0.10	20.5 ± 0.50
*Saccharomyces cerevisiae*	》	》	》	》	》

Note: 》: the whole plate had no colonies; the diameter of the inhibition zones (mm), including the filter paper diameter (6 mm), are given as mean ± SD of triplicate experiments.

## Data Availability

Data are included in the article.
